# ACTH-dependent Cyclic Cushing Syndrome With Successful Pregnancy and Early Postpartum Relapse

**DOI:** 10.1210/jcemcr/luaf079

**Published:** 2025-04-11

**Authors:** Mohd Idris Mohamad Diah, Jin Hui Ho, Hwee Ching Tee

**Affiliations:** Endocrinology and Diabetes Unit, Department of Medicine, Hospital Queen Elizabeth II, Kota Kinabalu 88300, Malaysia; Endocrinology and Diabetes Unit, Department of Medicine, Hospital Queen Elizabeth II, Kota Kinabalu 88300, Malaysia; Endocrinology and Diabetes Unit, Department of Medicine, Hospital Queen Elizabeth II, Kota Kinabalu 88300, Malaysia

**Keywords:** cyclic Cushing syndrome, ACTH-dependent Cushing syndrome, pregnancy, postpartum relapse

## Abstract

Cyclic Cushing syndrome (CS) is a rare form of CS characterized by intermittent episodes of hypercortisolism. We report the case of a 30-year-old female who was diagnosed with ACTH-dependent Cushing disease, confirmed by initial biochemical tests and pituitary imaging. Although surgery was planned, she experienced spontaneous remission for several months, followed by pregnancy, and subsequently relapsed in the early postpartum period. Transsphenoidal resection of a left-sided pituitary adenoma was then performed, confirming an ACTH-secreting tumor. A review of the literature revealed that this case contributes to the increasing number of patients with cyclic CS, with particular attention to the challenges of diagnosing hypercortisolism during pregnancy. While cases of Cushing disease recurrence after pituitary surgery in the immediate postpartum period have been documented, this is the first reported case of early postpartum relapse in cyclic CS. This case highlights the importance of long-term follow-up in patients with a high index of suspicion for cyclic CS, as well as the diagnostic challenges in managing the condition during pregnancy and the peripartum period.

## Introduction

Cyclic Cushing syndrome (CS) is an uncommon form of CS characterized by alternating phases of biochemical hypercortisolism (peaks) and normal or low cortisol levels (troughs) [1]. The cycles can present as either regular or irregular and may vary significantly in duration, ranging from days to months or even years, with “true cyclicity” defined as trough cortisol levels falling below the upper limit of normal [1, 2]. Additionally, diagnosing cyclic CS during pregnancy is even more challenging due to the overlap of its symptoms with normal pregnancy, which is associated with physiological hypercortisolism [3]. We present a rare case of ACTH-dependent cyclic CS with spontaneous conception during eucortisolemic phase, followed by a postpartum relapse.

## Case Presentation

**Figure 1.**
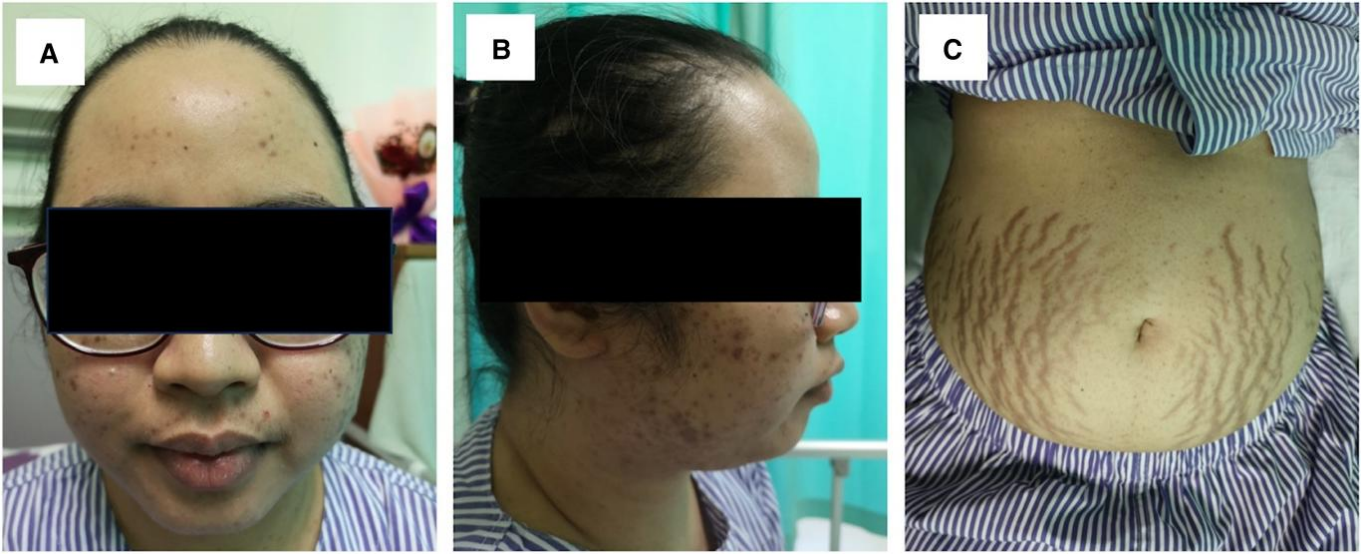
Clinical findings of Cushing syndrome during presentation in our case. (A) Round face; (B) acne with acanthosis nigricans; (C) red-purple striae on the abdomen.

A 30-year-old woman with no prior known medical conditions presented to our emergency department with a 2-week history of persistent headache and blurred vision. She also reported a progressive weight gain of 12 kg over the past year, accompanied by irregular menses. Upon arrival in the emergency room, she appeared cushingoid, with a blood pressure of 208/121 mmHg and a heart rate of 150 beats per minute. Physical examination revealed facial plethora, acne, a dorsocervical and supraclavicular fat pad, and skin thinning with purplish striae over the abdomen (Fig. 1). Other systemic examinations were unremarkable. She was newly diagnosed with diabetes mellitus and hypertension at the time of presentation.

## Diagnostic Assessment

Several endocrine investigations were conducted, suggesting ACTH-dependent Cushing disease. Serum cortisol following a low-dose 1 mg overnight dexamethasone test (ODST) was not suppressed at level of 279 nmol/L (10.1 μg/dL) (cutoff value 50 nmol/L [1.8 μg/dL]), and the 24-hour urinary free cortisol (UFC) level was markedly elevated at 2343 nmol/24 hour (849.3 µg/24 hours) (reference value 11.8-485.6 nmol/24 hours) (approximately 4.28-176.0 µg/24 hours). Additionally, the ACTH level was elevated at 14.7 pmol/L (66.9 pg/mL) (reference value 1.6-13.9 pmol/L [7.2-63.3 pg/mL]).

**Figure 2.**
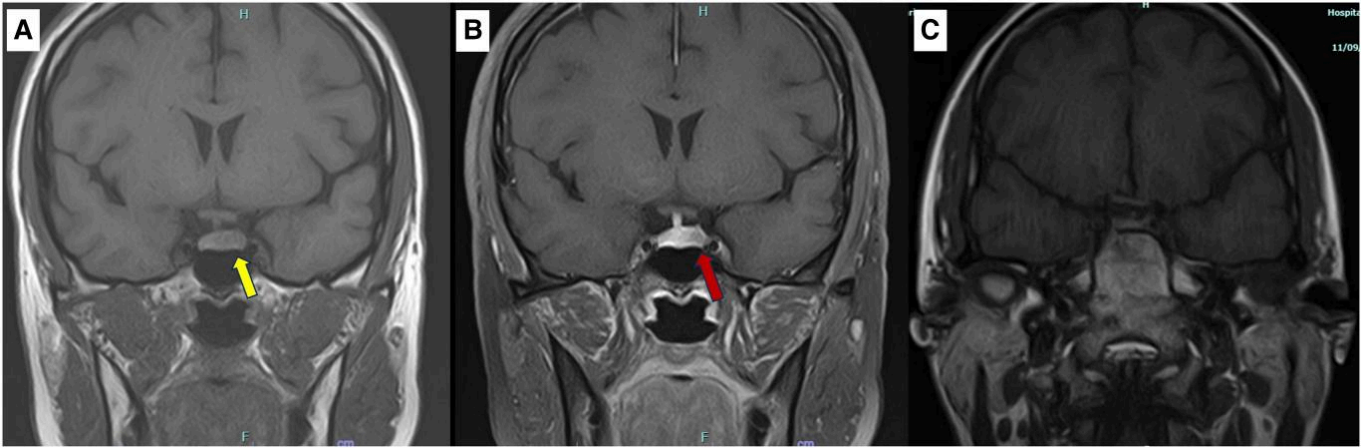
Coronal T1-weighted MRI pituitary at diagnosis and after transsphenoidal surgery. (A) A hypointense lesion on the T1-weighted image in the left anterior pituitary region, measuring 0.6 × 0.5 × 0.5 cm (yellow arrow). (B) The lesion exhibited minimally delayed enhancement following gadolinium contrast administration (red arrow). (C) Follow-up MRI 6 months after transsphenoidal surgery. Abbreviation: MRI, magnetic resonance imaging.

A pituitary magnetic resonance imaging (MRI) was performed to localize the source of excess cortisol, revealing a small, well-defined nodule with delayed enhancement in the left anterior pituitary gland measuring 0.6 × 0.5 × 0.5 cm (Fig. 2A and 2B). The optic chiasm, bilateral cavernous sinus, and posterior pituitary bright spot were intact.

Given the small lesion (less than 10 mm), inferior petrosal sinus sampling was conducted, which indicated a central source of ACTH secretion with lateralization to the left side, consistent with the pituitary MRI findings of a left pituitary microadenoma. She was started on oral antihypertensive and glucose-lowering medications while awaiting pituitary surgery.

**Figure 3.**
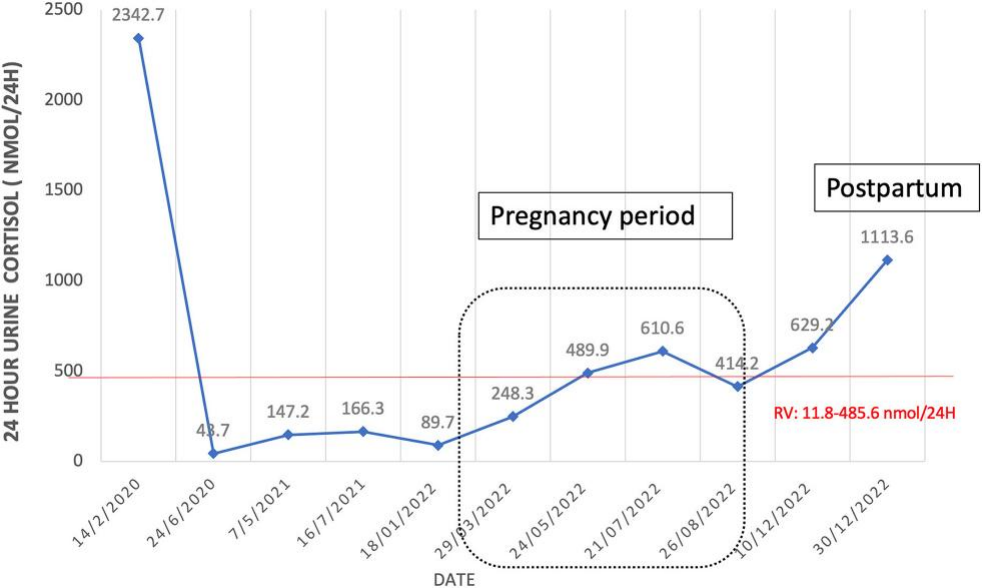
Timeline changes of 24-hour urinary cortisol level over the years. Normal limit reference value for 24-hour urine cortisol: 11.8 to 485.6 nmol/24 hours.

At her 4-month follow-up, she experienced a spontaneous complete resolution of cushingoid features, and her repeat 24-hour UFC returned to normal levels. Over the following 2 years and 4 months, her 24-hour UFC levels fluctuated between normal and the upper limit of normal (see Fig. 3). A low-dose 2 mg dexamethasone suppression test over 48 hours during this period showed a suppressed serum cortisol level with a value of 22 nmol/L (0.8 µg/dL) (cutoff value 50 nmol/L [1.8 µg/dL]), indicating normal cortisol secretion.

Notably, she had a spontaneous conception during the eucortisolemic phase. Although her 24-hour UFC levels were slightly elevated during the second and third trimesters, they remained below 3 times the upper normal limit, and she did not exhibit any cushingoid features throughout the pregnancy. Her antihypertensive and antidiabetic medications were gradually tapered off. During the third trimester, her blood pressure increased, necessitating the reintroduction of a single antihypertensive medication (oral methyldopa 250 mg 3 times a day). She subsequently developed preeclampsia, which required an emergency lower-segment cesarean section. Fortunately, the surgery was uncomplicated, and she gave birth to a healthy baby boy weighing 3.26 kg at 37 weeks.

However, 8 weeks after delivery, she began to redevelop signs of CS, including facial plethora, acne, purplish abdominal striae, and excessive weight gain. Repeat testing confirmed a relapse of endogenous hypercortisolism, with a 24-hour UFC level of 1113 nmol/24 hours (403.3 µg/24 hours) and an unsuppressed serum cortisol level of 118.9 nmol/L (4.3 µg/dL) after ODST, suggesting hypercortisolemia, with an elevated ACTH level of 11.4 pmol/L (51.7 pg/mL). A repeat MRI showed a slightly larger sellar lesion in the left anterior pituitary region measuring 0.8 × 0.6 × 0.5 cm.

## Treatment

**Figure 4.**
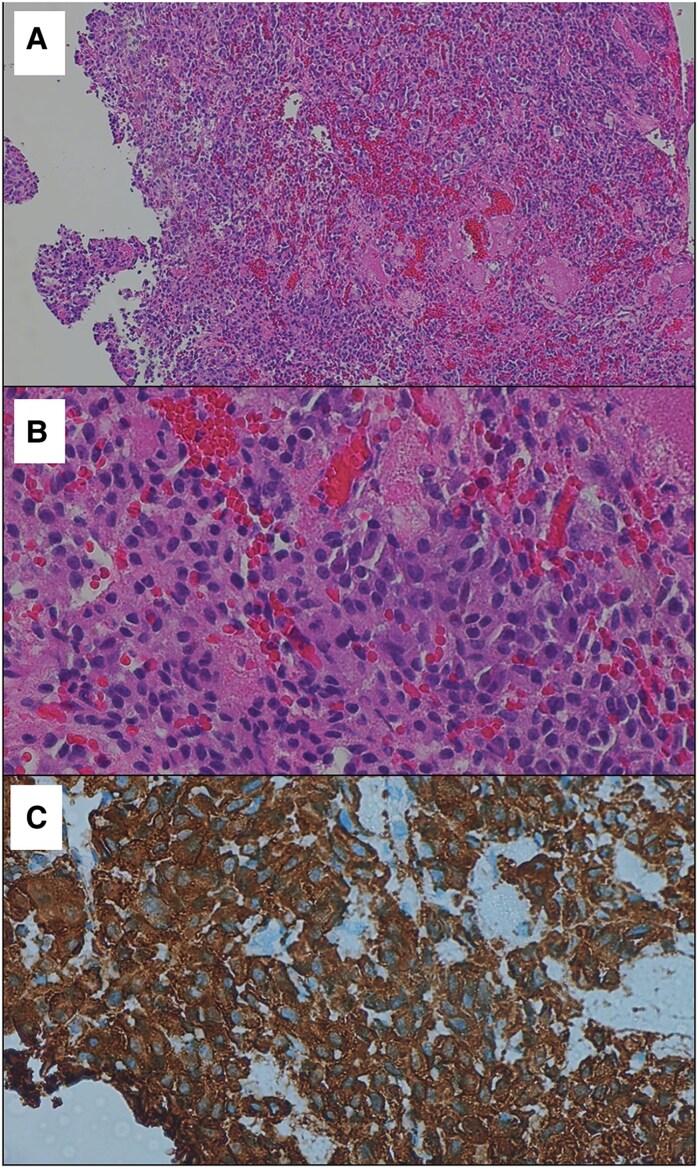
ACTH-secreting pituitary adenoma. (A) Tumor cells arranged in solid sheets (low magnification, H&E stain). (B) Cytologically, the tumor cells have uniform round nuclei, stippled chromatin texture, inconspicuous nucleoli, and a modest to ample amount of basophilic cytoplasm (high magnification, H&E stain). (C) The tumor cells express strong and diffuse cytoplasmic reactivity for ACTH on immunohistochemistry. Abbreviation: H&E, hematoxylin and eosin.

The patient subsequently underwent transsphenoidal resection of the pituitary microadenoma. Pathological findings were suggestive of a pituitary adenoma, with immunohistochemical staining showing positivity for synaptophysin and chromogranin. Further staining for ACTH confirmed an ACTH-secreting pituitary tumor. (Figure 4A-4C).

## Outcome and Follow-up

She required hydrocortisone replacement for 2 months following pituitary surgery. A follow-up MRI of the pituitary, performed 6 months postsurgery, showed no residual adenoma (Fig. 2C). At her most recent clinic visit, 18 months after surgery, she had lost 11 kg and displayed no features of hypercortisolaemia. Additionally, she no longer required antihypertensive or antidiabetic medications. All biochemical assays assessing her hormonal axes at 1 year postsurgery were within normal limits including a midnight salivary cortisol of <3 nmol/L (0.11 µg/dL) (cutoff value <11.3 nmol/L [0.41 µg/dL]) on 2 occasions.

## Discussion

Cyclic CS is a rare and challenging variant of CS, distinguished by intermittent periods of hypercortisolism. Unlike the more typical continuous cortisol elevation seen in noncyclic cases, patients with cyclic CS experience fluctuations in cortisol levels, leading to alternating phases of hypercortisolism and eucortisolism or even hypocortisolism. Two widely accepted diagnostic criteria for cyclic CS are the presence of either 2 peaks and 1 trough or 3 peaks and 2 troughs in cortisol levels. The prevalence of cyclic CS ranges from 14% to 18%, depending on which diagnostic criteria are applied [2, 4]. ACTH-secreting pituitary adenomas are the leading cause of cyclic CS, followed by ectopic tumors and adrenal causes, with the latter primarily involving micronodular adrenal hyperplasia or primary pigmented nodular adrenocortical disease. Pulmonary and thymic neuroendocrine tumors are the most frequently identified ectopic sources [2, 5]. In this case, the cyclic pattern was attributed to an ACTH-secreting pituitary microadenoma, confirmed histologically after transsphenoidal resection.

The diagnosis of cyclic CS is particularly challenging due to its intermittent nature. Clinical features typically manifest during hypercortisolemic phases and may resolve entirely during trough periods, as observed in our patient. She initially presented with classic symptoms of CS during a hypercortisolemic phase, followed by a prolonged eucortisolemic phase lasting approximately 2 years and 4 months. During this period, her symptoms gradually resolved completely, and her comorbidities, including hypertension and diabetes, were well controlled. The patient's spontaneous remission during the eucortisolemic phase raised the possibility of pituitary apoplexy as a potential cause. However, she did not exhibit clinical features suggestive of apoplexy, such as sudden headache, visual disturbances, or neurological deficits. Although an MRI was not performed during this phase to definitively rule out apoplexy, subsequent imaging showed no evidence of hemorrhage or infarction, and the pituitary microadenoma remained stable in size.

She experienced spontaneous conception during the trough period, which made monitoring challenging. The clinical features of CS during pregnancy can overlap with those of non-Cushing pregnant women, presenting similar symptoms such as weight gain, fatigue, edema, hypertension, and hyperglycemia. This overlap is due to the hyperactive hypothalamic-pituitary-adrenal axis in pregnancy, where elevated estrogen levels increase the production of corticosteroid-binding globulin, leading to higher circulating cortisol. Additionally, placental CRH stimulates maternal ACTH and cortisol secretion, mimicking the hypercortisolism seen in CS and complicating the diagnosis [6, 7].

The Endocrine Society's practice guidelines recommend using 24-hour UFC and late-night salivary cortisol (LNSC) measurements to best indicate cyclicity [8]. In cases where initial tests return normal results despite strong clinical suspicion, follow-up with repeat testing, preferably timed with the appearance of symptoms, is advised. Scalp hair cortisol concentration is another method that has been reported as an additional tool for assessing long-term cortisol exposure and may aid in detecting cyclicity in CS [9]. ODST results may be normal in patients who are cycling out of hypercortisolism.

In our case, we used UFC to assess hypercortisolemia since LNSC and hair cortisol concentration were unavailable at our center at the initial period. The UFC test demonstrates good sensitivity and specificity for diagnosing CS when the upper limit of normal is applied as a criterion [8, 10]. However, it is important to note that UFC excretion typically increases 2- to 3-fold during the second and third trimesters as a normal physiological response. Therefore, in suspected cases of CS during pregnancy, only UFC levels greater than 3-fold above the upper reference limit may suggest active CS, aiding in the differentiation from the normal rise in cortisol associated with pregnancy [11]. During the second and third trimesters, our patient showed elevated 24-hour UFC levels, but they were still below 3 times the upper limit of normal. However, the development of preeclampsia near delivery raised suspicion of an early relapse, which was confirmed postpartumly.

The patient experienced a recurrence of hypercortisolism 8 weeks after delivery, highlighting the potential for disease relapse in the postpartum period. A retrospective study by Tang et al reported that 27% of premenopausal women in their cohort developed Cushing disease related to pregnancy, suggesting a possible link between pregnancy and disease recurrence [12]. However, evidence supporting postpartum relapse remains limited, and the underlying mechanisms are not fully understood. Hypercortisolism in the immediate postpartum period may be triggered by the transient but substantial surge in ACTH and cortisol levels during labor, particularly in susceptible individuals. Other proposed mechanisms include hormonal changes during pregnancy, such as elevated estrogen levels, which can stimulate pituitary angiogenesis, hormone secretion, and tumor growth via estrogen receptors α and β expressed in corticotroph adenomas. Postpartum estrogen withdrawal or residual tumor activation may further contribute to relapse [12-14].

There is currently no consensus on defining remission criteria following tumor resection in patients with cyclic CS. In this case, the patient is considered in remission following pituitary surgery, as evidenced by the absence of a detectable pituitary mass on imaging and postoperative adrenal insufficiency requiring glucocorticoid replacement. Prolonged hypercortisolism prior to surgery often leads to suppression of corticotroph cells, resulting in low levels of ACTH and cortisol postoperatively. Differentiating true remission from a temporary trough phase in cyclic CS requires careful and ongoing clinical, biochemical, and imaging assessments. True remission is often characterized by postoperative adrenal insufficiency, necessitating temporary glucocorticoid replacement, as observed in this case. However, the hallmark of cyclic CS is fluctuating cortisol levels, and sustained normal cortisol levels must be confirmed through regular serial testing to distinguish true remission from a transient trough phase.

**Figure 5.**
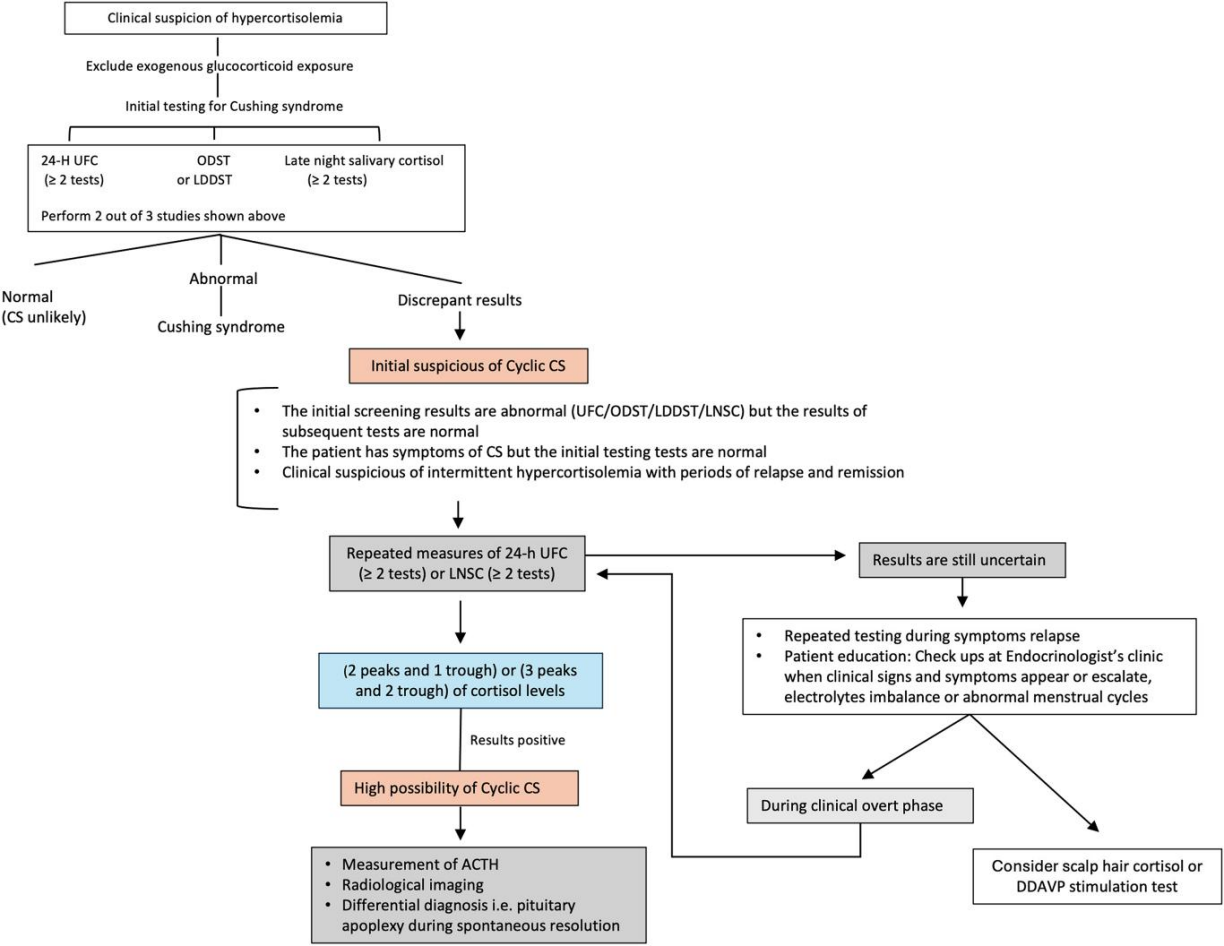
Proposed diagnostic algorithm for suspected cyclic Cushing syndrome.

We proposed an algorithm for cases with a high suspicion of cyclic CS (Fig. 5).

## Learning Points

- Close monitoring and repeat testing are essential for patients with a high suspicion of cyclic CS, given the intermittent nature of hypercortisolism.
- The 24-hour UFC test is useful for screening CS during pregnancy and is 1 of the best indicators of cyclicity in suspected cyclic CS, alongside the LNSC test.
- Relapse can occur in the immediate postpartum period in patients with cyclic CS, necessitating careful observation.

## Data Availability

Original data generated and analyzed during this study are included in this published article.

## Abbreviations

CS: Cushing syndrome
LNSC: late-night salivary cortisol
MRI: magnetic resonance imaging
ODST: overnight dexamethasone test
UFC: urinary free cortisol
